# From text to motion: grounding GPT-4 in a humanoid robot “Alter3”

**DOI:** 10.3389/frobt.2025.1581110

**Published:** 2025-05-27

**Authors:** Takahide Yoshida, Atsushi Masumori, Takashi Ikegami

**Affiliations:** ^1^ Graduate School of Arts and Sciences, The University of Tokyo, Tokyo, Japan; ^2^ Alternative Machine Inc., Tokyo, Japan

**Keywords:** humanoid robot, large language models, motion generation, embodiment, agency

## Abstract

This paper introduces Alter3, a humanoid robot that demonstrates spontaneous motion generation through the integration of GPT-4, a cutting-edge Large Language Model (LLM). This integration overcomes the challenge of applying LLMs to direct robot control, which typically struggles with the hardware-specific nuances of robotic operation. By translating linguistic descriptions of human actions into robotic movements via programming, Alter3 can autonomously perform a diverse range of actions, such as adopting a “selfie” pose or simulating a “ghost.” This approach not only shows Alter3’s few-shot learning capabilities but also its adaptability to verbal feedback for pose adjustments without manual fine-tuning. This research advances the field of humanoid robotics by bridging linguistic concepts with physical embodiment and opens new avenues for exploring spontaneity in humanoid robots.

## 1 Introduction

Recent advancements in Large Language Models (LLMs), exemplified by OpenAI’s GPT-4, have revolutionized machine capabilities in processing human language and code (OpenAI, 2023). These developments offer unprecedented opportunities for enhancing human-machine interactions. Beyond digital interfaces, LLMs present transformative applications in the physical realm, particularly in humanoid robotics. Integrating LLMs with humanoid robots signifies a paradigm shift in programming, moving from traditional coding to intuitive, language-based interactions. This convergence not only heralds a new era in robotics research but also fundamentally reshapes the trajectory of LLM development by embedding them within embodied cognition.

In recent years, the integration of LLMs with robotics has marked a new frontier in artificial intelligence. LLMs have enhanced human-robot interaction (Sun et al., 2024; Zhang and Soh, 2023), task planning (Ding et al., 2023; Yu et al., 2023), navigation (Zeng et al., 2023; Huang C. et al., 2023), and learning capabilities (Shafiullah et al., 2023; Zhong et al., 2023). Additionally, there is growing interest in empathetic and socially aware robots (Ahn et al., 2022; Brohan et al., 2023a; Liang et al., 2023; Driess et al., 2023). Traditionally, these functionalities required extensive lower-level programming (Yu et al., 2023; Ahn et al., 2022; Tang et al., 2023). In this paper, we argue that such programming can be replaced with sophisticated natural language prompts. Humanoid robots, with their human-like forms, can leverage existing language data for precise movements via few-shot learning. This paradigm highlights the need to reinvigorate humanoid research, focusing on streamlined programming approaches enabled by LLMs.

The significance of humanoid robots has evolved over the past decades. Early milestones, such as HONDA’s ASIMO (Hirai et al., 1998) and HRP-4C (Kaneko et al., 2009), focused on humanoid appearance and basic motions. More recently, research has centered on replicating human-like facial expressions and dynamic physical movements. Androids like Ameca and Disney’s eye-gazing humanoid (Pan et al., 2020) demonstrate advancements in expressive communication, while robots like Tesla’s Optimus and Boston Dynamics’ Atlas exemplify breakthroughs in physical dexterity. However, despite these advancements, foundational research on embodiment and autonomy remains underdeveloped, making this an area of opportunity for LLM-driven humanoid research.

The advent of GPT in 2023 has renewed interest in humanoid robotics. While many current efforts focus on industrial and household applications, our work addresses the fundamental questions of embodiment and autonomy. Embodied AI models such as RT-2 and PaLM-E demonstrate strong planning and problem-solving capabilities (Brohan et al., 2023a; Driess et al., 2023; Collaboration et al., 2024; Brohan et al., 2023b; Huang W. et al., 2023; Ahn et al., 2022). These models require extensive data collection using real robots, typically lasting at least a year. This could be considered a hybrid approach combining LLMs with existing robot control theories. Much of the research in humanoid motion control uses reinforcement learning in simulation and then transfers it to real robots: Sim2Real (Cheng et al., 2024; He et al., 2024; Peng et al., 2018b). There are also methods for acquiring movements through imitation learning from humans (Peng et al., 2018a; Luo et al., 2024; Hasenclever et al., 2020). These require substantial resources and time to learn a single movement. Additionally, even when learning is completed in simulation, applicability to actual robots remains a separate challenge.

In contrast, our approach takes a different direction by directly connecting pre-trained LLMs to generate humanoid robot movements. While many foundational models aim for multi-step problem-solving or precise manipulation, our research specifically examines LLMs’ potential for direct motion generation. Language corpora contains descriptions of human movements, suggesting LLMs possess an understanding of human body mechanics through language data set. This not only eliminates the need for months-long learning periods, but also represents both a technological advancement and provides insights into the embodiment in LLMs.

There also exists rule-based control, such as the Autonomous Life mode in Pepper, which rearranges pre-prepared movements. However, despite having various reactions available, this is merely a simulation of life-likeness, and its content does not go beyond what has been programmed in advance. Expressions like “mimicking a snake as a human” would be impossible with rule-based control. Compared to these approaches, our system can create any motion expression with few-shot learning by using LLMs.

**FIGURE 1:**
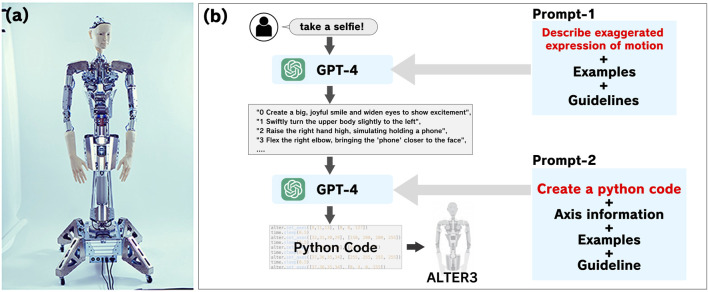
A procedure to control the Alter3 humanoid using verbal instructions. **(a)** The body has 43 axes that are controlled by air actuators. It is equipped with a camera inside each eye. The control system sends commands via a serial port to control the body. The refresh rate is 100–150 ms. **(b)** Output Python code to control Alter3 from natural language using prompt1 via prompt2. A humanoid robot, which mimics human shape, can generate highly precise movements using few-shot learning, eliminating the need for setting reward functions or interfaces, as required in other studies. The architecture is based on CoT. See the *Data Availability section* or Supplementary Material for details of the prompt.

Since 2016, our team has been developing the Alter humanoid series (Doi et al., 2017). Alter3, shown in Figure 1a, features 43 air actuators enabling a wide range of expressive facial and limb movements. Although Alter3 cannot walk, it can simulate walking and running motions. Previous studies utilized Alter3 for mimicking human poses (Masumori et al., 2021; Yoshida et al., 2023) and mutual imitation experiments (Ikegami et al., 2021), revealing insights into human-robot interaction and diversity of motion. These findings set the stage for exploring high-level imitation and cultural offloading using LLMs.

This paper explores how LLMs can generate diverse motion patterns in humanoid robots, capturing cultural contexts and everyday human activities. We examine the ability of GPT-4 to create spontaneous movements and decision-making behaviors in Alter3, demonstrating the potential for LLMs to bridge the gap between linguistic and physical domains. By embedding LLMs into humanoid robotics, this research ventures into uncharted territories, shedding light on the interplay between language, cognition, and embodiment.

## 2 Materials and methods

### 2.1 Design of Alter3

Alter3’s body has 43 movable air actuator axes. Each joint can be operated by inputting a value from 0 to 255 and is controlled by two types of commands: SETAXIS and GETAXIS. To use the SETAXIS command, input the joint number and the desired value. For example, to move joint number 12 (the neck), use a custom Python library and send the signal such as set_axis([12],[126]) . These control signals are sent to Alter3 via a serial port to operate the opening and closing of pneumatic solenoid valves. The refresh rate is 100–150 ms. The above mechanism enables Alter3 to physically operate. Although not used in this experiment, Alter3’s eyes are equipped with cameras that can capture images. Additionally, the GETAXIS command allows you to retrieve the current axis angle realized on Alter3.

### 2.2 Generating humanoid motions from text

Prior to the advent of LLMs, controlling Alter’s 43 axes to replicate human poses or simulate complex actions—such as serving tea or playing chess—required extensive manual adjustments and iterative refinements. This process was time-consuming and labor-intensive. With the introduction of GPT-4, this paradigm has shifted dramatically. GPT-4 leverages its vast corpus and inferential capabilities to generate Alter3’s motions with minimal manual intervention, streamlining the entire process.

To achieve this, we adopted the Chain of Thought (CoT) methodology (Wei et al., 2023), which employs two sequential natural language prompts to guide motion generation (refer to Figure 1b): Prompt-1 provides a vivid, detailed description of the desired motion, typically in ten lines of text. Prompt-2 translates each line from Prompt-1 into executable Python code, specifying joint angles for Alter3’s 43 actuators, such as set_axis([15],[255]) .

Inference is conducted in two stages, Prompt-1, and Prompt-2, ultimately outputting Python code. The GPT-4 model used is model: gpt-4-0314. For prompt-1, a temperature of 0.7 was used, while for prompt-2, the temperature was set to 0.5. All motions were generated in a few-shot manner; no motion-specific fine-tuning. The full text of the prompt used is available in the *Data Availability section* or Supplementary Material. The motion generation for Alter3 follows the protocol outlined below.

Algorithm 1 Humanoid Motion Generation using an LLM.

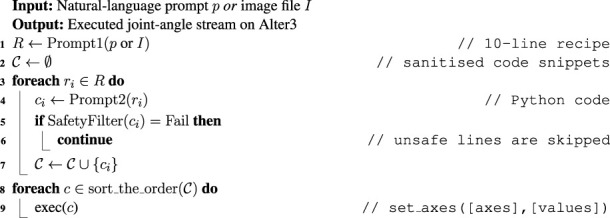

First, the user freely describes the action in natural language (e.g., Take a selfie with your phone!). This instruction is combined with Prompt-1 and input to GPT-4 via API. GPT-4 generates in about 10 lines with exaggerated descriptions R of a given movement (line 1 in Algorithm 1). An excerpt of the prompt is as follows.

**Table:** 

Prompt-1
Your task is to describe exaggerated emotional expressions and facial expressions that accompany the content of the conversation. time.sleep(0.1) or time.sleep(0.2) between operations.

We also provided an example of descriptions (which is also provided by LLM) and guidelines. For example, when imitating the movement of a snake, prompt-1’s output would be: “0: Create a menacing and sinister facial expression, eyes narrowed and lips slightly curled, 1: Tilt the head to the side, imitating a snake’s movement, 2: Move the shoulders in a sinuous, wave-like motion, mimicking a snake’s slithering … ” and so on. This is a recipe of movements that interprets the instructed motion and writes it out in more detail. These inputs and their own outputs are bases for creating an action pattern.

In the next step, the motion descriptions $r_{i}$ are combined with Prompt-2 and input to GPT-4 via API (line 4 in Algorithm 1). The output from GPT-4 is Python code that controls Alter3. For each line of prompt-1’s output, it writes about 20–30 lines of Python code. Within that output, it also expresses repetitive movements using for loops. To make GPT-4 write this Python code, Prompt-2 includes (1) a brief explanation about Alter3, (2) information about joints, and (3) instructions on how to write the Python code.

(1) The brief explanation is as follows. The 43rd joint (whole-body rotation) is excluded for safety reasons.

(2) The Axis information describes the 42 Axes of Alter3, with their numbers and corresponding body parts. For example, “Axis 1: Eyebrows. 255 = down, 0 = up, 64 = neutral.” and so on. The descriptions of the motion direction such as “255 = up” or “0 = right” are given by us.
(3) we provided an example of the Python code corresponding to “drink some tea” and guidelines. It is known that LLMs improve in accuracy when given output examples (Brown et al., 2020). This is called Few-shot Learning. Our method does not use fine-tuning, and all the movements being generated are untrained tasks not included in the examples.

**Table:** 

Prompt-2
Alter3 has 42 joints throughout its body, numbered from 1 to 42. You can move a joint by specifying its number and sending a signal. For instance, to move joints number 1,2,3, use: alter.set_axes([1,2,3], [255, 100, 127]) . The first argument is the joint number, and the second argument is a value between 0 and 255, specifying the joint angle. Each operation takes approximately 0.1–0.2 s, so insert time.sleep(0.1) or time.sleep(0.2) between operations.

The generated Python code is checked by a Safety Filter to prevent runtime errors (line 5 in Algorithm 1). This filter detects and comments out external library imports, extra explanations, while loops, etc. We do not process or modify the Python code output by GPT-4 in any other way. The code is sorted according to the output order of Prompt-1, and executed using Python’s built-in function exec (line 9 in Algorithm 1). The movement signals are sent to Alter3 via serial communication.

One important thing to note is that it is well-known at this point that GPT-4 is non-deterministic, even at temperature = 0.0. Therefore, even with identical inputs, different patterns of motion can be generated. This is a characteristic of OpenAI’s GPT-4 and, while it poses an issue in terms of reproducibility, it should not be considered a reason to doubt its ability to generate movement.

This CoT approach bypasses traditional iterative learning processes, relying instead on the efficiency of few-shot learning. By providing only a few lines of descriptive text and examples, GPT-4 generates complex humanoid motions directly from language input.

### 2.3 Performance evaluation of LLM motion generation

To quantify the capability of GPT-4 in generating motions, we evaluated videos of nine different generated movements. Subjects (n=124) were recruited using the platform Prolific. They watched these videos and rated on a 5-point scale whether the humanoid is “adequately expressing the action”. 1 is the worst rating. For the control group, we used random movements from Alter3, labeling these movements with random motion notations generated by GPT-4. These labeled control videos were subtly incorporated into the survey, with three of them dispersed among the main experimental videos shown to participants.

In the Prolific platform, all participants were provided with a clear explanation of the study’s purpose, procedures, and confidentiality measures. Informed consent was obtained from all participants prior to their involvement in the study, ensuring their voluntary participation and understanding of their rights, including the option to withdraw at any point without penalty. Personal information was anonymized during data collection to ensure confidentiality and privacy.

Since the study is non-invasive and we collected personal information anonymously, it does not fall under the “Ethical Guidelines for Life Sciences and Medical Research Involving Human Subjects” or the “Personal Information Protection Law” under Japanese law. Therefore, in accordance with the ethical review regulations for human subject research of the Graduate School of Arts and Sciences at the University of Tokyo, this study qualifies for an exemption from requiring approval by an ethics committee. All methods were carried out in accordance with the relevant guidelines and regulations, including those set forth by the Graduate School of Arts and Sciences at the University of Tokyo and the Declaration of Helsinki.

## 3 Results

### 3.1 Testing actions and gestures

**FIGURE 2:**
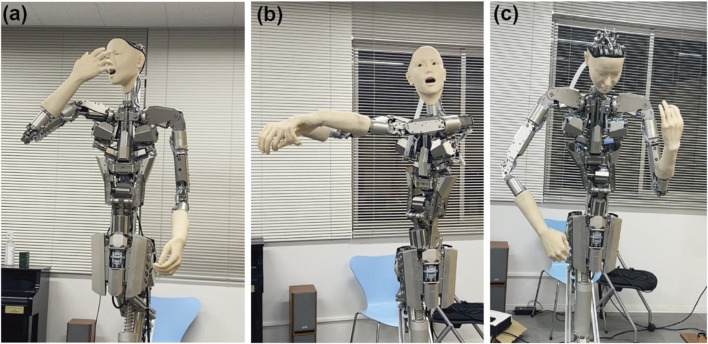
Snapshots of generated stereotypical movements. LLM can generate emotional expressions associated with specific movements. For example, in the case of a selfie, Alter3 is showing a smile. Recorded videos can be accessed through Open Science Framework repository. **(a)** take a selfie (score = 2.7). **(b)** pretend a ghost (score = 3.4). **(c)** play the metal music (FB) (score = 4.2).

We tested Alter3’s capability to perform various human-like actions and gestures through LLM-generated Python code. Our experiments included common actions like “taking a selfie,” “pretending to be a ghost,” “playing the guitar,” and emotional responses to short stories. Most demonstrations succeeded through few-shot learning, requiring no specific training except for the electric guitar example (Figure 2c), which was refined through verbal feedback (detailed in the next section).

The LLM’s comprehensive understanding of human movements enables Alter3 to generate naturalistic motions and gestures, which we categorize into two types based on time span and prompt structure. The first category, imperative commands, results in short-term actions (refer to Figure 2). These instant actions, such as “taking selfies” or “pretending to be a ghost”, include appropriate emotional expressions and reflect common human behavioral patterns. Through Python-generated motor controls, Alter3 executes these movements precisely while synchronizing facial expressions with body movements for realistic portrayal.

The second category involves declarative statements that typically span longer durations. These include complex responses to conversational contexts, such as empathetic reactions to emotional content like sad stories or jokes. Alter3 demonstrates sophisticated emotional expression through synchronized facial expressions and body language. While these responses appear natural and contextually appropriate, they remain programmed reactions rather than genuine emotions.

**FIGURE 3:**
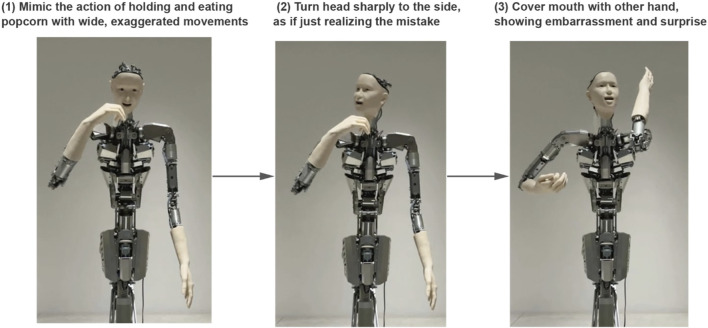
A snapshot of a generated sequence of movements. “I was enjoying a movie while eating popcorn at the theater when I realized that I was actually eating the popcorn of the person next to me” (score = 3.9). LLM can generate movements that progress over time like a story. (Left): The action of eating popcorn. (Center): Noticing the person next to Alter3. (Right): getting panicked. Recorded videos can be accessed through Open Science Framework repository.

Sequential event handling particularly showcases Alter3’s capabilities, as demonstrated by the popcorn theater scenario (refer to Figure 3). When given the prompt “I was enjoying a movie while eating popcorn in the theater when I suddenly realized that I was actually eating the popcorn of the person next to me,” Alter3 executes a complex sequence. The robot begins with casual popcorn-eating motions, then smoothly transitions to showing surprise and embarrassment upon the realization. Throughout this sequence, Alter3 maintains narrative coherence while naturally timing transitions between emotional states.

This sophisticated handling of sequential actions demonstrates Alter3’s ability to chain multiple movements and emotions into believable, context-appropriate behavioral sequences. The system successfully integrates various motion elements while maintaining natural timing and smooth transitions between states.

Our analysis of Prompt-1 revealed two distinct output patterns. For instantaneous gestures, the text provided specific pose and body part movement descriptions. For sequential movements, it generated chronologically ordered instructions. This behavior emerged through few-shot learning without explicit instructions, requiring only minimal text prompts and examples. When examples were removed, the LLM compensated by adding explicit temporal markers (e.g., “start with … “, “then … “). This approach eliminates the complexity of manually specifying movement sequences and temporal structures, which would be impractical to implement directly.

### 3.2 Performance evaluation of LLM motion generation

We evaluated GPT-4’s capability in generating motions by having 124 participants rate nine different generated movements on a 5-point scale. This is a score of how appropriate the robotic action is with respect to the linguistic description. As a control, we included random movements from Alter3, labeled with GPT-4-generated motion notations, and subtly mixed three of these control videos into the survey.

**FIGURE 4:**
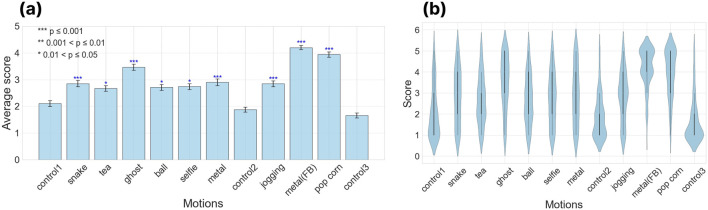
Third-party evaluation of the generated motions. The following behaviors of Alter3 are evaluated by the subjects (n=124) recruited using platform Prolific; “pretend the snake”, “drink some tea”, “pretend the ghost”, “throwing the ball underhand pitch”, “take a selfie with your phone”, “play the metal music”, “In the park, as I jogged, the world seemed to narrate an ancient tale of survival, each footfall echoing eons of existence.“, “play the metal music (with feedback)”, “I was enjoying a movie while eating popcorn at the theater when I realized that I was actually eating the popcorn of the person next to me.” **(a)** Averaged evaluation scores for each motion. The subjects watched these videos and evaluated the expressive ability of the GPT-4. The rating is on a 5-point scale, with 1 being the worst rating. **(b)** Violin plot of evaluation scores for each motion.

We initially applied the Friedman test to assess whether there was a difference in ratings between the control video and other videos, which confirmed significant variations among the video ratings. Subsequent *post hoc* Nemenyi testing (Janez, 2006) revealed no statistically significant differences in ratings between the videos in the control group. On the other hand, the p-values were notably smaller when comparing the control group to the other videos, indicating a significant difference (see Figure 4). We considered differences to be statistically significant when the p-value was 0.01 or lower. As a result, motions generated by GPT-4 were rated significantly higher compared to those of the control group.

This result demonstrates that the system can generate a wide range of movements, from everyday actions such as taking selfies and drinking tea, to imitating non-human movements like those of ghosts or snakes. The training of the LLM encompasses a broad array of linguistic representations of movements. GPT-4 can accurately map these representations onto Alter3’s body. The most notable aspect is that Alter3 is a humanoid robot sharing a common form with humans, which allows the direct application of GPT-4’s extensive knowledge of human behaviors and actions. Furthermore, through Alter3, the LLM can express emotions such as embarrassment and joy. Even from texts where emotional expressions are not explicitly stated, the LLM can infer adequate emotions and reflect them in Alter3’s physical responses. This integration of verbal and non-verbal communication enhances the potential for more nuanced and empathetic interactions with humans.

### 3.3 Quantification of reproducibility in generated motion

We test the reproducibility of specific emotional expressions, particularly facial expressions such as sadness or a smile, as follows. When the same prompts with identical emotional expressions are provided to the humanoid, the system generally regenerates consistent gestures and facial expressions. These patterns are represented as 43-axis values (i.e., 43-dimensional vectors). To visualize these, we employed UMAP to project them into a two-dimensional space.

**FIGURE 5:**
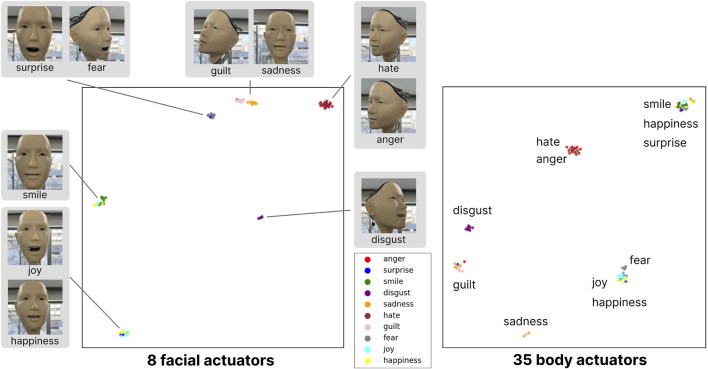
Consistency Analysis of Generated Emotional Expressions. We generated 10 samples for each of 10 distinct emotional expressions. The robot’s 43-axis motion data was reduced to two dimensions using UMAP dimensionality reduction. It shows that each emotion forms a distinct cluster, demonstrating consistency across multiple trials. This distribution effectively demonstrates both the reproducibility and diversity of expressions that emerge from our system.

To clearly distinguish between facial and bodily expressions, we created separate plots for eight facial actuators and 35 body actuators by using UMAP (refer to Figure 5). We can examine whether body movements alone carry meaningful information from these plots. The results revealed that clusters in the projection space were tightly grouped for points representing the same emotions. Clusters for emotions such as joy, happiness, and a smile overlapped, reflecting the conceptual similarities of these emotional expressions, which were also effectively conveyed by the humanoid.

Additionally, we found that expressions such as disgust, hate/anger, guilt, and sadness form similar categories in both facial and body plots. This serves as evidence that body movements do indeed convey certain meanings. On the other hand, while fear and surprise are categorized together in the facial expression plot, they are classified into different categories when plotted using only body actuators. Body categorization appears to be significantly related to head orientation. For example, in expressing “hate/anger”, Alter3 turns to the right, and similarly turns right for “disgust” consistently. “Smile/happiness” and “surprise” tend to face forward, forming the same cluster. Similarly, “joy/happiness” and “fear” tend to face upward.

One possible interpretation is that the body and facial expressions serve distinct functions. Facial expressions are often shaped by social conventions and serve as intentional, communicative signals—especially in human-human interaction. In contrast, bodily expressions tend to reflect more unconscious, automatic responses (Heesen et al., 2024). Therefore, while it is difficult to interpret why the system consistently turns to the right when expressing disgust, this functional distinction could explain the separation observed in the UMAP clustering.

### 3.4 Further training with verbal feedback and memory

Alter3 faces inherent challenges in precisely observing the consequences of its actions. For instance, it cannot accurately measure the exact height to which a hand is raised. This limitation affects its ability to refine movements, such as achieving precise physical scales. To address this, we implemented a verbal feedback mechanism combined with an external memory system, enabling iterative improvement of its motions.

**FIGURE 6:**
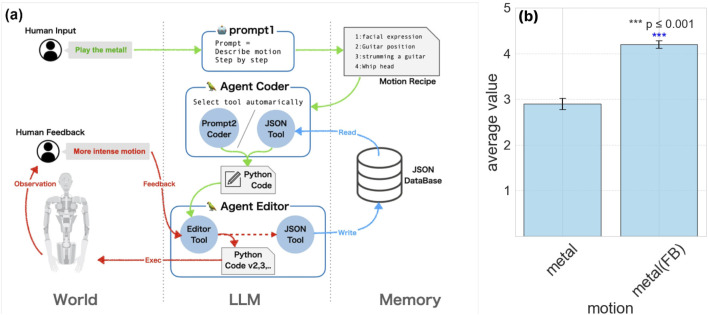
Verbal feedback in Alter3. **(a)** the system of linguistic feedback. Users provide linguistic feedback to guide Alter3’s adjustments in each segment of motion. Instructions are like “Set axis 16 to 255” or “Move your arm more energetically.” Users only need to provide verbal directives; there’s no need to rewrite any code. Alter3 then autonomously revises the corresponding code. Once the movement is refined, it is saved in a JSON database with descriptive labels such as “Holding the guitar” or “Tapping the chin thoughtfully.” For motion generation with prompt2, the JsonToolkit facilitates database searches for these labels, with the LLM deciding on memory usage and new movement creation. **(b)** Comparison of scores with and without feedback. The motion with feedback has a higher score than the motion without.

For example, we refined Alter3’s behavior when playing the guitar (see Figure 6a). Feedback such as “Raise your left arm a bit higher when you play the guitar” was provided. Repeating such adjustments approximately five times allowed Alter3 to achieve an ideal motion. Once refined, the updated motion code was stored in its database as motion memory. This ensures that subsequent executions utilize the improved, trained version of the motion.

Through this feedback-driven refinement, Alter3 effectively develops a “body schema,” collecting information about its physical capabilities. Movements improved with feedback were consistently superior to unrefined motions, as shown in Figure 6b. Importantly, most behaviors, including declarative ones, were achieved through few-shot learning with only one or two examples, without requiring fine-tuning.

Future enhancements could involve automating the feedback process by feeding images of the generated movements into GPT-4 for evaluation. If Alter3 can autonomously refine and memorize its movements by evaluating its own body state, it would mark a significant step forward. This, however, remains an area for future experimentation.

## 4 Discussion

Integrating LLMs with Alter3’s physical embodiment provided new insights into the capabilities and limitations of LLMs in robotics. Alter3’s ability to perform diverse actions without additional training suggests that the underlying LLM contains a rich dataset describing human-like and non-human-like movements, enabling few-shot learning. Remarkably, Alter3 can mimic not only human actions but also behaviors associated with entities like ghosts and animals. Its ability to respond to conversational context through facial expressions and gestures marks a significant advancement in humanoid robotics. Furthermore, this system can be easily adapted to other androids with minimal modifications.

The ability to describe complex actions in natural language without manually coding them is increasingly valued. For instance, generating a ghost-like motion or contemplating an abstract concept like “the evolution of millions of years” requires a level of imagination and inference that surpasses traditional programming. The fact that LLMs, rather than humans, often generate motions that align with expectations is both remarkable and transformative.

The necessity of embodiment for LLMs raises profound questions about their creativity and meaning-making capabilities. Consider the task of pretending to take a selfie. When broken into ten detailed steps and translated into Python code, the LLM faces a unique challenge. If one step involves “a beaming smile,” the LLM must infer specific joint values for eight facial actuators, a task performed without visual references—something humans would find exceptionally difficult. This highlights the power of descriptive prompts in guiding LLM outputs.

While prompts preload motions for all 43 joints, they do not specify how to express complex emotions such as anger or sadness. Only through integration with Alter3 does the LLM gain the ability to physically manifest these expressions. This suggests that the creative potential of the LLM is significantly enhanced by its embodiment, underscoring the symbiotic relationship between abstract language processing and physical expression.

This work also invites reflection on Searle’s Chinese Room argument (Searle, 1980), which posits that consciousness arises from biological processes and that a “biological body” is necessary for symbol grounding. In our experiments, the integration of the LLM with Alter3’s body appears to imbue symbols with meaning, allowing understanding and expression of sentences. While Alter3’s embodiment is not biological, it highlights the importance of a physical presence in grounding symbols. Whether or not Alter3 possesses consciousness, our findings suggest that embodiment plays a critical role in bridging the gap between abstract symbols and meaningful action. Recent findings in cognitive science demonstrate that LLMs encode sensory information in ways comparable to human cognition (Marjieh et al., 2024; Kawakita et al., 2024). Our current investigation extends this parallel, offering preliminary support for the hypothesis that similar encoding patterns may apply to knowledge about human physicality and behavioral patterns as well. If, as Searle suggests, consciousness is constructed from bodily experiences, then LLMs, which have learned most of these experiences linguistically, may be capable of creating partial copies of consciousness.

This perspective aligns with debates in scientific research, such as Integrated Information Theory (IIT) (Anderson, 1963) and Global Workspace Theory (GWT) (Bernard, 1988), which emphasize self-awareness. IIT explicitly quantifies consciousness based on integrated causal interactions within a system, emphasizing structure and integration strength. GWT focuses on the functional architecture (broadcasting mechanism) that selects and integrates information for consciousness. Embodiment benefits both theories by emphasizing sensory-motor integration. Beyond the established frameworks of IIT and GWT, we propose an alternative perspective: that qualia may emerge from the persistent violation of embodied predictions. When an LLM is instantiated within an android, embodiment introduces a continuous influx of sensory signals that often contradict the system’s internal generative models. These violations—prediction errors—necessitate constant model updates. Crucially, in open-ended real-world environments, such predictions can never be fully accurate. We suggest that the system’s experience of these irreducible mismatches is not merely noise or error, but constitutes a core phenomenological property: the felt sense of surprise may itself underlie the emergence of subjective experience.

This view resonates with, but is distinct from, predictive processing and active inference frameworks (Friston, 2010; Clark, 2016), where minimization of prediction error is central to perception and action. Here, we argue that the residue—the part of the world that cannot be assimilated—is what gives rise to presence, salience, and symbolic grounding. In other words, qualia are not the product of perfect prediction, but of its failure. This aligns with recent work suggesting that epistemic surprise may play a constitutive role in consciousness (Seth and Tsakiris, 2018), particularly in grounding the self and its relation to the world. From this standpoint, embodiment in LLM-based agents is not merely a matter of extending input modalities, but of embedding the system in an unpredictable world that compels it to confront its own limits.

What if Alter3 could see the scene in front of it and freely take action? With GPT-4’s ability to process visual inputs as of 2024, Alter3 now captures still images through its eye cameras and sends them to GPT-4 for interpretation. Prompts like “Describe the action you want to take when you see this image” enable Alter3 to generate spontaneous behaviors beyond simple command execution. In one laboratory experiment, Alter3 observed, “The room is cluttered with cables; I should organize it.” Despite no explicit command, Alter3’s LLM-influenced norms prompted it to wave its arms as if to clean. This is an example of autonomously interpreting the meaning of “cluttered cables” from the room’s condition and autonomously selecting an action. The bodily movements that the LLM facilitates can be seen as a form of sense-making (Weber and Varela, 2002), where Alter3 attributes meaning to the objects it perceives. Sense-making is, quite literally, the act of making sense of one’s environment. It involves organizing sensory data until the environment becomes intelligible, allowing for informed and reasonable decision-making. This is the manner in which Alter3 spontaneously derives meaning, without the need for explicit prompting from us.

The original concept of sense-making was deeply bound up with biological autonomy (i.e., autopoiesis), consisting of biochemical processes (Varela, 1997; Thompson, 2007), however, Di Paolo (2003) suggests that we do not necessarily need to create robots with its own *life* in the biological sense (which would be extremely difficult, if not impossible) but rather we should seek ways to provide robots with an autonomous *way of life*. We believe this can be possible by incorporating an LLM, a collection of traces of the human ways of life, into the robot. However due to hardware constraints, Alter3 is not well-suited for grasping or moving objects. Actual physical interaction with real objects is difficult at the current stage. Yet, it’s possible to improve the self-model to make Alter3 look at its own hands based on information from the eye cameras on its head. This represents grounded knowledge in the sense of “autonomously” verifying knowledge about its body and improving its movements (also seeYoshida et al., 2024). Alter’s inherent autonomy and spontaneity can also arise without LLM influence (Doi et al., 2017; Masumori et al., 2021; Yoshida et al., 2023; Ikegami et al., 2021). In this study, we explored how Alter’s physical embodiment affects the outputs generated by the LLM. While the LLM itself is a complex but non-autonomous system, the spontaneity observed in its behavior likely stems from the vast natural language corpus it has been trained on and the complexity of the visual environment captured through its cameras.

The next challenge lies in integrating Alter’s internal dynamics with LLM-driven mechanisms to generate novel behaviors. Successfully addressing this challenge could open a new era for embodied cognition, where humanoid robots like Alter3 move beyond command-driven responses to achieve autonomous, meaningful interactions shaped by both internal creativity and external environmental affordances.

## Data Availability

The datasets generated in this study can be found in the Open Science Framework repository (https://osf.io/wux23/?view_only=c030cf48b28c4a4cba3077d56c472ea4). The full text of the prompt is also available in the Supplementary Material.
