# Supplementary material for: From text to motion: grounding GPT-4 in a humanoid robot “Alter3”
Source: Front Robot AI. 2025 May 27;12:1581110. doi: 10.3389/frobt.2025.1581110 (PMC12149125; doi:10.3389/frobt.2025.1581110)
Supplement: Supplementary file 4 [file DataSheet1.pdf]

Supplementary Material

1 PROMPTS FOR MOTION GENERATION

This is the details of the prompts used for humanoid motion generation. In Prompt-1, the desired actions (e.g., serving tea, playing chess) are vividly described over several lines, capturing each step and clearly outlining the overall motion.

Prompt-1

Your task is to describe exaggerate emotional expressions and facial expressions that accompany the content of the conversation. Output motion description should be several simple motions that the android is capable of. In addition, please create a facial expression that matches the input at the beginning. The android can only move its upper body and has the same joints as a human. Output should be written as much detail as possible.
Example1:
input : [”Driving to Hokkaido sounds like a great idea, Julia. The landscapes there could provide some great inspiration for my art.”]
description :
[”Showing excitement about driving to Hokkaido, several exaggerated motions are below”,
”0 Create a wide-eyed facial expression of thrill and anticipation”,
”1 Swiftly lean forward, dramatically showing interest”,
”2 Raise both hands high and spread them out widely to emphasize the idea”,
”3 Place one hand on the heart, indicating deep emotional connection to the thought”,
...
]
Example 2:
input : [”drink some tea”]
description :
[
”drink some tea, several simple motions is below”,
”0 Create a peaceful and content facial expression, eyes slightly narrowed in a relaxed manner”,
”1 Turn towards cup”,
”2 Reach for cup”,
”3 Grasp cup”,
...
]
Guidelines:
1: Output should be list.
2: Write as much detail as possible. Describe action step by step. Do not write any explanation.Describe exaggeration as much as possible.
3: Android has only upper body.
4: Create a facial expression that matches the input at the beginning.
input :{input}

Prompt-2 takes each descriptive line and translates it into executable Python code. Specifically, each line is mapped to a joint angle command for Alter3's 43 axes (e.g., setaxis([15], [255])).

Prompt-2

Write python code to operate an android named Alter3. Here's what you need to know.
Alter3 has 42 joints throughout its body, numbered from 1 to 42. You can move a joint by specifying its number and sending a signal. For instance, to move joints number 1,2,3, use: alter.setaxes([1,2,3], [255, 100, 127]). The first argument is the joint number, and the second argument is a value between 0 and 255, specifying the joint angle. Each operation takes approximately 0.1 second, so insert time.sleep(0.5) between operations.
Alter3's Joints:
- Axis 1: Eyebrows. 255 = up, 0 = down, 64 = neutral.
- Axis 2: Pupils (horizontal). 255 = left, 0 = right, 140 = neutral.
- Axis 3: Pupils (vertical). 255 = up, 0 = down, 128 = neutral.
- Axis 4: Eyes. 255 = closed, 0 = open.
- Axis 5: Left cheek. 255 = raised, 0 = lowered.
- Axis 6: Right cheek. 255 = raised, 0 = lowered.

...
Example:drink some tea
```
# Face the front with the cups
alter.setaxes([13, 11, 9], [150, 150, 255])
time.sleep(0.5)
# Reaching for a virtual tea cup
alter.setaxes([29, 32, 30, 31], [200, 150, 150, 200])
time.sleep(0.5)
...
```
Task:
Your task is to write a python code that causes Alter to perform the following actions.
Input is a description of the sequence of movements. The format is ”motion : description”.
Movements should be lengthened or shortened depending on the content of the input.
Based on this, write the movement commands for Alter3. The Axis value is between 0 and 255
The output is just the code, no explanation is required.DO NOT insert python.
Guidelines:
1: Output should be only python code. Do not insert any syntax highlighting.
2: Do not insert python syntax highlighting.
3: Do not write “import alter”.
4: Use # and write short description of code.
action :{input}

2 MOTION INSTRUCTIONS VIA NATURAL LANGUAGE

The method of modifying Alter3 motions generated by natural language consists of the following steps.

- Step 1: As previously mentioned, generate motion from language through prompt1 and prompt2. The generated code is divided into code blocks corresponding to each line of the output from prompt1.

- Step 2: After observing the executed motion, a human input the segments and guidance in natural language. Instructions are like "Set axis 16 to 255" or "Move your arm more energetically." Users only need to provide verbal directives; there's no need to rewrite any code. Alter3 then autonomously rewrites the corresponding code. Repeat this step until the human is satisfied.
- Step 3: After the movement has been fine-tuned, it gets stored in a JSON database with descriptive tags like "Holding the guitar" or "Tapping the chin thoughtfully."

3 FRIEDMAN TEST AND NEMENYI TEST

The Friedman test is a non-parametric statistical test used to check for differences in the average ranks among three or more matched groups with ordinal data. The null hypothesis in a Friedman test is that there are no differences in the medians across all groups. The p-value of given data $\{x_{ij}\}_{n,k}$ is derived as follows. In this study, n is the number of subjects ($n = 124$), and k is the number of generated motion ($k = 9$) Firstly, find the values

$$T_j = \sum_{i}^{n} R_{ij} \quad (j = 1, 2, 3, \dots, k)$$

Where R_ij is rank the evaluated scores for each subject. Calculate the Friedman $F + r$ statistic using the formula:

$$F_r = \frac{12}{nk(k+1)} \sum_{j}^{k} T_j^2 - 3n(k+1)$$

Finally, compare F_r to the chi-square distribution and obtain p-value. If the null hypothesis is rejected following the Friedman test, a post-hoc test such as the Nemenyi test is conducted. The Nemenyi test involves performing pairwise comparisons between all groups to evaluate whether there are statistically significant differences in each comparison. We use the scipy library to conduct the Friedman test and the Nemenyi test.
